# Local prostate cancer radiotherapy after prostate-specific antigen progression during primary hormonal therapy

**DOI:** 10.1186/1748-717X-7-209

**Published:** 2012-12-10

**Authors:** Michael Pinkawa, Marc D Piroth, Richard Holy, Victoria Djukic, Jens Klotz, David Pfister, Axel Heidenreich, Michael J Eble

**Affiliations:** 1 Department of Radiation Oncology, RWTH Aachen University, Pauwelsstrasse 30, 52072 Aachen, Germany; 2 Department of Urology, RWTH Aachen University, Pauwelsstrasse 30, 52072 Aachen, Germany

**Keywords:** Prostate cancer, Radiotherapy, Brachytherapy, Ir-192, Prostate-specific antigen, Hormone therapy

## Abstract

**Background:**

The outcome of patients after radiotherapy (RT) for localized prostate cancer in case of prostate-specific antigen (PSA) progression during primary hormonal therapy (HT) is not well known.

**Methods:**

A group of 27 patients presenting with PSA progression during primary HT for local prostate cancer RT was identified among patients who were treated in the years 2000–2004 either using external-beam RT (EBRT; 70.2Gy; n=261) or Ir-192 brachytherapy as a boost to EBRT (HDR-BT; 18Gy + 50.4Gy; n=71). The median follow-up period after RT was 68 months.

**Results:**

Median biochemical recurrence free (BRFS), disease specific (DSS) and overall survival (OS) for patients with PSA progression during primary HT was found to be only 21, 54 and 53 months, respectively, with a 6-year BRFS, DSS and OS of 19%, 41% and 26%. There were no significant differences between different RT concepts (6-year OS of 27% after EBRT and 20% after EBRT with HDR-BT).

Considering all 332 patients in multivariate Cox regression analysis, PSA progression during initial HT, Gleason score>6 and patient age were found to be predictive for lower OS (p<0.001). The highest hazard ratio resulted for PSA progression during initial HT (7.2 in comparison to patients without PSA progression during primary HT). PSA progression and a nadir >0.5 ng/ml during initial HT were both significant risk factors for biochemical recurrence.

**Conclusions:**

An unfavourable prognosis after PSA progression during initial HT needs to be considered in the decision process before local prostate radiotherapy. Results from other centres are needed to validate our findings.

## Background

External beam radiotherapy (EBRT) and temporary interstitial brachytherapy (HDR-BT) are all well established radiotherapy (RT) techniques for a curative treatment of localized prostate cancer [1-7]. The combination with hormonal therapy (HT) has been shown to be associated with improved overall survival for high risk patients after EBRT in several prospective randomized studies [8-12]. HT is frequently administered before brachytherapy for downsizing the prostate volume [13].

The optimal sequencing and duration of HT in combination with EBRT is not well known. A longer duration of HT proved to be associated with an overall survival benefit for high risk patients in randomized trials addressing this question [9,10,14]. A difference was even found for a neoadjuvant HT comparing eight versus three months [14].

In daily practice, patients sometimes do not receive a well defined short-term neoadjuvant HT before presenting in the radiotherapy department. In some cases, definitive curative treatment is only postponed. Patients sometimes receive HT until PSA levels rise after an initial PSA nadir. These patients were in focus of this analysis.

Patients after EBRT alone and EBRT with an additional HDR-BT boost have been included in this evaluation to verify the results independently in two separate patient groups with different radiotherapy concepts. Randomized trials comparing these concepts with two different dose prescriptions have shown improved biochemical relapse-free survival applying an HDR-BT boost. However, survival rates were not reported to differ significantly [15,16].

## Methods

A group of 27 patients presenting with PSA progression during primary HT for local prostate cancer RT was identified among patients who were treated in the years 2000–2004 either using external-beam RT (EBRT; 70.2Gy; n=261) or Ir-192 brachytherapy as a boost to EBRT (HDR-BT; 18Gy + 50.4Gy; n=71). The indication for a specific treatment was generally based on the patient’s and/or the referring urologist’s preference. Only 7 patients received a short-term neoadjuvant HT <6 months. The median follow-up period after RT was 68 months.

A bone scan and abdominal computed tomography scan was required to exclude lymph node or skeletal metastases for high risk patients at the time of the initial diagnosis.

### Treatment

The referring urologist decided about the indication for HT due to prognostic risk factors or to offer an immediate treatment before a later decision for a definitive curative method. As a consequence, several different agents have been used: luteinizing hormone-releasing hormone (LHRH) agonists in 10 cases (37%), antiandrogens in 3 cases (11%), a combination of LHRH agonists and antiandrogens in 5 cases (19%) and an orchiectomy in 9 cases (33%) in the group with PSA progression during initial HT.

An Ir-192 stepping source from an afterloader with a nominal activity of 370 GBq was used for temporary HDR-BT. All patients received two fractions to deliver 18Gy to the prostate with 7 days between each fraction. Within three weeks after brachytherapy EBRT started. Three dimensional treatment plans were calculated using a four-field box technique with 15MeV photons and a multi-leaf collimator. The planning target volume was required to be enclosed by the 90% isodose relative to the International Commission on Radiation Units and Measurements reference point with a margin of 1.5cm in the anterior/lateral and 1cm in the craniocaudal and dorsal directions to the clinical target volume (prostate +/− seminal vesicles). The total median dose to the prostate in the reference point was 50.4Gy at 1.8Gy daily fractions. For EBRT without additional brachytherapy, the same technique was used up to a median dose of 70.2Gy at 1.8Gy fractions.

### Follow-up

All patients had a pretreatment PSA measurement. PSA data since the initial diagnosis were collected retrospectively from the referring urologist, including the start of HT and PSA nadir value. The PSA levels were usually obtained every 3 or 4 months in the first 2 years and every 6 months thereafter. PSA failure (biochemical failure) was defined according to the RTOG-ASTRO Phoenix consensus [17]: (1) a rise by 2 ng/ml or more above the nadir PSA; (2) the date of failure determined “at call” (not backdated). An initiation of hormonal treatment after RT was additionally counted as biochemical failure.

Patients who died were censored at the time of death according to their status at that time. Disease specific mortality was defined as death from prostate cancer (patients who died with evidence of disease recurrence without an independent diagnosis responsible for the patient’s death). Overall mortality was defined as death from any cause.

### Statistical analysis

Statistical analysis was performed using the SPSS 19.0 (SPSS, Chicago, Ill), software. Contingency table analysis with the chi-square test was performed to compare treatment groups with respect to categorical variables. A t-test was used to compare patient age, follow-up periods and initial PSA values for patients in different subgroups.

Kaplan-Meier analysis was used to determine biochemical recurrence, disease specific and overall survival. Comparisons between groups were made using the log-rank test. Prognostic factors (T stage, Gleason score, pre-treatment PSA), radiotherapy technique, HT, duration of initial HT (dichotomized in >6 months = “long-term”, and ≤6 months = “short-term”), PSA progression during initial HT (PSA levels reaching a nadir and rising before beginning of RT) and PSA nadir before RT after initial HT (last PSA before beginning of RT considered for analysis) were tested for their significance in a univariate and multivariate Cox regression analysis (p<0.05 is considered significant).

## Results

Nearly half of the total patient group (n=149) received an initial HT before RT with a curative intent, 19% (n=62) for a time of more than 6 months (median 15 months, range 6–165 months). A PSA progression during initial HT was found in 8% (27 patients; 8%, n=22, in the EBRT and 7%, n=5, in the HDR-BT subgroup).

Baseline patient characteristics for patients presenting with PSA progression during primary HT in contrast to other patients treated with one of the mentioned RT concepts in the years 2000–2004 are presented in Table 1. Patients with PSA progression during primary HT are clearly a selection of patients with adverse prognostic factors already at the time of the initial diagnosis.

**Table 1:** Baseline patient characteristics

	**PSA progression during initial HT (n=27)**	**Patient population without PSA progression (n=305)**
patient age/years median (range) mean±SD	74 (45–81) 72±8	71 (52–84) 71±6
follow-up period/months† median (range) mean±SD	48 (11–113) 50±23	69 (2–115) 66±18
T stage >2a†	74%	25%
Gleason score >6†	48%	19%
primary PSA /ng/ml† median (range) mean±SD	16(4–150) 31±37	10(1–300) 16±25
low risk patients^*^†	7%	37%
intermediate risk patients^**^†	7%	28%
high risk patients^***^†	85%	35%
initial HT (before RT)†	100%	40%
initial HT >6months†	78%	13%
HDR-BT	19%	22%
EBRT	81%	78%

^* ^no risk factors: PSA<10ng/ml; Gleason score<7; cT-stage<2b.

^** ^one risk factor: PSA 10-20ng/ml or Gleason score=7 or cT-stage=2b/c.

^*** ^two risk facors or PSA>20ng/ml or Gleason score>7 or cT-stage>2b/c.

†statistically significant difference (p<0.01).

*Abbreviations: *HDR-BT = high-dose rate brachytherapy; EBRT = external beam radiotherapy; SD = standard deviation; PSA = prostate-specific antigen; HT = hormonal therapy.

Median biochemical recurrence free (BRFS), disease specific (DSS) and overall survival (OS) for patients with PSA progression during primary HT was found to be only 21, 54 and 53 months, respectively, with a 6-year BRFS, DSS and OS of 19%, 41% and 26%. There were no significant differences between different RT concepts (6-year OS of 27% after EBRT and 20% after EBRT with HDR-BT). Metastases were diagnosed in 16 patients, with the initial location in bones in 11 patients and lymph nodes in 6 patients.

The outcome after EBRT and HDR-BT was far more favourable if all 332 patients were considered (6-year BRFS: 60% vs. 57%; 6-year DFS: 91% vs. 93%; 6-year OS: 77% vs. 78%; median values not reached). HDR-BT tended to be superior to EBRT alone for low risk patients considering BRFS (6-year BRFS: 94% vs. 72%; p=0.08), with excellent DFS rates (6-year DFS: 100% vs. 99%). A lower PSA nadir after initial HT was found to be predictive for BRFS (6-year BRFS: 89% vs. 50% with a nadir ≤0.5 ng/ml vs. >0.5 ng/ml; p=0.006). PSA progression during initial HT was a crucial adverse prognostic factor (6-year OS with vs. without PSA progression: 26% vs. 84%; p<0.001; 6-year DFS with vs. without PSA progression: 41% vs. 94%; p<0.001).

**Figure 1:**
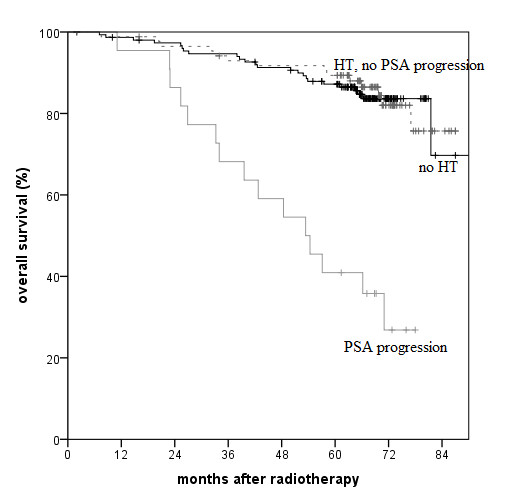
**Overall survival after EBRT as single modality. **(p<0.001 comparing PSA progression during initial HT vs. no PSA progression/no HT in log-rank test).

**Figure 2:**
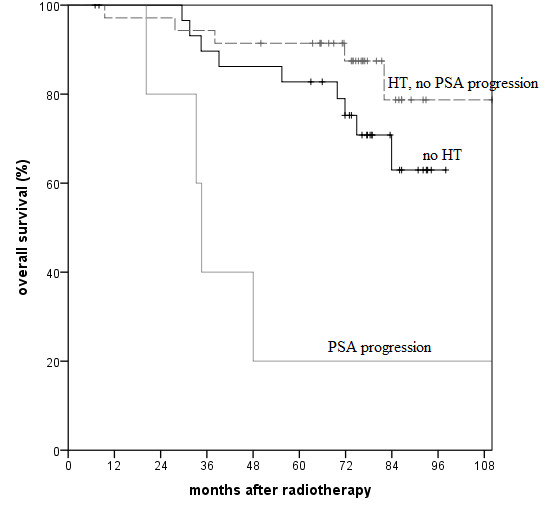
**Overall survival after EBRT with additional HDR-BT boost. **(p<0.001 comparing PSA progression during initial HT vs. no PSA progression; p=0.005 comparing PSA progression vs. no HT in log-rank test).

Only a few factors proved to have a significant impact in the multivariate analysis (Table 2). The established factors T stage >2a, Gleason score >6 and PSA >10 ng/ml were all of prognostic relevance. Only Gleason score was found to be significantly relevant for (disease specific and overall) survival in our patient population. PSA progression and a nadir >0.5 ng/ml during initial HT were both significant risk factors for biochemical recurrence. However, additionally to the Gleason score and patient age, only PSA progression during HT remained a significant predictor for survival in the multivariate analysis. Figures 1 and 2 well demonstrate the prognostic impact of PSA progression independently for the patient groups after EBRT alone or EBRT with an additional HDR-BT boost.

**Table 2:** Significant factors in multivariate analysis

	**Hazard ratio**	**95% confidence interval**	**P-value**
**Biochemical recurrence free survival**			
T stage ≤2a	1.7	1.1-2.5	0.014
PSA <10ng/ml	1.9	1.2-2.8	0.003
no PSA progression vs. PSA pr. with HT	3.4	1.9-6.0	<0.001
no PSA progression vs. no HT	1.5	1.2-21	0.024
nadir ≤0.5 vs. >0.5 after initial HT	4.7	1.1-19	0.034
**Disease specific survival**			
Gleason score ≤6	4.0	1.8-9.1	0.001
no HT vs. PSA progression with HT	23	7.3-72	<0.001
no PSA progression vs. PSA pr. with HT	9.1	3.7-22	<0.001
**Overall survival**			
age <70years	2.6	1.5-4.4	<0.001
Gleason score ≤6	2.2	1.4-3.7	0.002
no HT vs. PSA progression with HT	5.2	3.6-9.4	<0.001
no PSA progression vs. PSA pr. with HT	7.2	3.9-14	<0.001

*Abbreviations: *PSA = prostate-specific antigen; HT = hormonal therapy.

**Figure 3:**
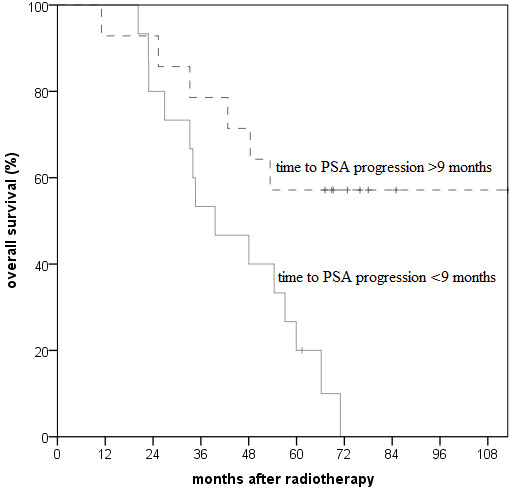
**Overall survival, stratified by the time to PSA progression after starting hormonal therapy. **(p=0.01 comparing time to PSA progression >9 months vs. ≤9 months in log-rank test).

Patients with a shorter time to PSA progression after starting HT were found to have particularly low overall survival rates (Figure 3; 6-year overall survival of 0% vs. 57% with time to PSA progression ≤9 months vs. >9 months). Median time to PSA progression was 9 months (in 41% <6 months). Patients with a shorter time to PSA progression (≤9 months) tended to have more frequently higher Gleason scores (62% vs. 36% Gleason score >6) and T stages (85% vs. 64% >2a).

Most of the patients with PSA progression during initial HT started RT with a PSA level <10 ng/ml (63%). OS of patients with PSA progression and a PSA level <10 ng/ml vs. ≥10 ng/ml (last value before starting RT) did not differ significantly.

## Discussion

Antiandrogen hormonal therapy is frequently combined with different RT methods. Treatment concepts are often based on the results of randomized trials, particularly for high risk patients receiving EBRT as a single modality [9-11]. A longer duration of HT is usually favoured for high risk patients according to the results of prospective randomized trials, especially considering an *adjuvant* HT. Studies from the EORTC (European Organization for Research and Treatment of Cancer) and RTOG (Radiation Therapy Oncology Group) could demonstrate the survival benefit of the longer HT duration (three and two years) in comparison to a shorter duration (four and six months) [9,10]. A secondary analysis of the RTOG 85–31 study reported improved survival for patients with HT treatment duration of more than five years (in comparison to one to five or less than one years) [18].

In contrast to HT, dose escalation studies did not show an overall survival benefit yet. A meta-analysis of randomized, controlled trials reported overall survival rates of 86% for both high-dose and low-dose radiotherapy [19], so that a relatively low total dose of 70.2Gy for patients treated with EBRT alone in this study can not explain a prognostic disadvantage in this respect. Accordingly - corresponding to the results of this study - randomized studies comparing a HDR-BT boost to EBRT in comparison to EBRT alone did not result in an overall survival benefit [15,16].

Even six months of short-term *neoadjuvant* HT proved to be associated with increased overall survival in locally advanced prostate cancer (TROG, Trans-Tasman Radiation Oncology Group, 96.01 trial) [20]. A Canadian multi-center trial comparing three months versus eight months of *neoadjuvant* HT in patients with localized prostate cancer has shown a significant overall survival benefit for high risk patients [18]. However, a recently published analysis of this trial found the biochemical response to neoadjuvant HT to be the critical determinant of benefit in the setting of combined therapy. Multivariate analysis identified post-hormone PSA (PSA nadir before beginning of RT), Gleason score, initial PSA and T-stage, not HT duration, as independent predictors of biochemical disease free survival [21]. The PSA level after 7 months of HT has also been found to be a strong independent predictor of survival in new metastatic prostate cancer in a Southwest Oncology Group (SWOG) trial [22]. These results are in accordance with the results of our study, demonstrating the independent impact of prognostic factors and post-hormone PSA nadir on biochemical disease free survival. An additional independent prognostic risk factor was found in our patient population, namely a PSA progression during initial HT.

With a significant impact for biochemical recurrence free survival in multivariate analysis, the PSA nadir could probably become significant in multivariate analysis for disease and overall survival after a longer follow-up interval. Patients with PSA progression during initial HT appear to be a selection with extremely aggressive prostate cancer. These cancers have a considerable impact on short term survival rates in contrast to usually expected high disease specific survival rates after definitive curative RT (99% 5-year DSS for patients without initial HT in this study).

Two possible reasons can be attributed to this unfavourable prognosis. First, these tumours consist of a considerable amount of preselected aggressive cells that are resistant to antiandrogen therapy and apparently simultaneously resistant to radiation. Secondly, these tumours have a high metastatic potential, leading to the imminent threat to the patient’s life. A particularly unfavourable prognosis was found for patients with a PSA progression after only a few months of HT, indicating a fast resistance to treatment.

A comparable study of patients receiving irradiation for localized prostate cancer in case of a PSA progression after initial HT is presently not available in the literature. The survival rates that were found in our study after RT are comparable to studies evaluating the outcome of androgen independent prostate cancer without any recorded local treatment [23,24]. Svatek et al. [24] reported a median disease specific and overall survival of 54 months and 51 months, respectively, for a population of 129 untreated consecutive patients with androgen independent prostate cancer. These data are well comparable to a median disease specific and overall survival of 54 months and 53 months in our study, so that a benefit of a local RT is not clear in this patient population. The decision for a local RT should be made considering this prognosis.

This is a retrospective analysis in a consecutively treated patient population, so that limitations exist due to a variety of underlying confounding factors. The generated hypothesis should be evaluated in other independent data sets. Imaging studies, like computed tomography of the abdomen, bone scan or choline-PET [2] might be useful not only at the time of the initial diagnosis but also before the decision for local RT even with smaller PSA levels, taking into account the high metastatic potential.

## Conclusions

PSA progression during initial antiandrogen hormonal therapy was found to be independently associated with an unfavorable prognosis after local radiotherapy for prostate cancer. This outcome needs to be considered in the decision process before local prostate radiotherapy. However, results from other centres are needed to validate our findings.

## Competing interests

The authors declare that they have no competing interests.

## Authors’ contributions

MP, MJE have made substantial contributions to conception and design; MP, MDP, RH, VD, JK, MJE have made substantial contributions to acquisition of data; MP, MDP, RH, DP, AH, MJE to analysis and interpretation of data. MP has been involved in drafting the manuscript. MDP, RH, VD, JK, DP, AH, MJE revised it critically for important intellectual content. All authors have given final approval of the version to be published.
